# Macrophage activation syndrome triggered by coeliac disease: a unique case report

**DOI:** 10.1186/s12969-016-0128-y

**Published:** 2016-12-09

**Authors:** J. Palman, J. May, C. Pilkington

**Affiliations:** 1 University College London Great Ormond Street Institute of Child Health, London, UK; 2 Great Ormond Street Hospital, London, UK

**Keywords:** Macrophage activation syndrome, Haemophagocytic lymphohistiocytosis, Coeliac disease, Autoimmune disease

## Abstract

**Background:**

Macrophage activation syndrome is described as a “clinical syndrome of hyperinflammation resulting in an uncontrolled and ineffective immune response” in the context of an autoinflammatory or rheumatic disease. Current associations of macrophage activation syndrome with autoimmune disease most notably include a host of rheumatological conditions and inflammatory bowel disease. Epidemiological studies have shown that macrophage activation syndrome is precipitated by autoimmune disease more commonly than previously thought. Diagnosing the precipitating factor is essential for effective treatment and prognosis.

**Case presentation:**

We report a case of a six year old girl with coeliac disease diagnosed after two episodes of secondary haemophagocytic lymphohistiocytosis. Her condition only responded to treatment once the patient was placed on a gluten free diet. Further immunological testing confirmed anti-transglutaminase and anti-endomysial antibodies, however histological biopsy was deemed inappropriate due to the severity of her condition. She has remained stable with no further episodes of macrophage activation syndrome since commencing a gluten free diet.

**Conclusion:**

This case report is the first literature that links macrophage activation syndrome to coeliac disease and highlights the challenge of diagnosing coeliac disease with unusual features such as associated prolonged fever. Clinicians should have a low threshold for screening children with other autoimmune diseases for coeliac disease.

## Background

Macrophage activation syndrome (MAS) is a serious complication of autoimmune disease with epidemiological reports of 4.2% in known cases of Juvenile Idiopathic Arthritis (JIA) and Systemic Lupus Erythematosus (SLE) [1]. Multiple reports have described MAS complicating a number of other autoimmune and auto-inflammatory diseases such as Kawasaki Disease [2, 3], Periodic fever syndromes [4] and inflammatory bowel disease [5, 6]. The significant mortality associated with the condition makes early diagnosis and early management important. Even with treatment using high dose steroids and biologics, patients with MAS complicating systemic-onset JIA (SoJIA) required intensive care in 34.9% and had an associated mortality of 8.1% [7]. A number of epidemiological studies have shown that MAS may be more common in autoimmune disease than previously thought [8–10].

Haemophagocytic lymphohistiocytosis (HLH) is defined by the 2004 criteria as molecular manifestations of known genetic defects (such as in perforin gene or Munc 13–4 gene) and clinical features. [11] MAS has a slightly different definition to HLH by not have a genetic component, as illustrated by the 2016 classification in SoJIA [12]. The presentation of MAS can often mimic the underlying systemic condition, with a rapid onset of persistent fever, reduced cognitive state, hepatosplenomegaly, lymphadenopathy, liver dysfunction and haemorrhagic skin manifestations. These features are found in conjunction with a fall in at least two haematological lineages, resulting in anaemia, thrombocytopenia or neutropenia, caused by an increased consumption and phagocytosis of the cells in the bone marrow.

The pathology behind MAS has been suggested by a number of studies as the immune mediated process resulting in secondary HLH. Some authors use the terms synonymously whilst others have defined MAS as a subtype of secondary HLH [13–15]. Primary HLH incorporates familial HLH (fHLH) and HLH caused by genetic immune deficiencies such as Chediak-Higashi and X-linked lympoproliferative disorder [14]. Differentiating between the primary and immunological cause is important for management options and prognosis. In particular, a diagnosis of primary disease carries the burden of treatment ending in haemopoeitic stem cell transplant and genetic counselling, compared to secondary disease which requires management of the underlying autoimmune process [16, 17].

The pathophysiology underlying macrophage activation syndrome is considered to be an excessive activation of mature macrophages and T-lymphocytes [18]. These macrophages infiltrate the bone marrow and inactivate cytotoxic T-cell function and natural killer cells, leading to an extreme overproduction of uncontrollable pro-inflammatory cytokines. Studies have correlated clinical symptoms and biological markers of MAS to an overproduction of IL-18 and the imbalance between IL-18 and IL-18 binding protein (the natural inhibitor) [19, 20]. Studies have also found IL-10 and IFN-γ have important roles in disease pathophysiology and severity of MAS [21, 22].

Coeliac Disease (CD) is an immune mediated condition precipitated by gluten and gluten products in patients with a genomic predisposition of HLA-DQ2 or HLA-DQ8. The condition is confirmed by having anti-tissue transglutaminase (anti-tTG) antibodies and anti-endomysium antibodies (EMA) or histological evidence of duodenal inflammation, crypt hyperplasia and villous atrophy [23]. Strict criteria for the diagnosis of CD has been made by European Society for Paediatric Gastroenterology, Hepatology and Nutrition (ESPGHAN) as diagnosing a patient carries a lifelong gluten free diet. The criteria also outlines the necessary screen for symptomatic patients when a biopsy is not performed, *i.e.* significant anti-tTG and anti-EMA on a background of HLA-DQ2/8 positivity [24]. For IgA anti-tTG and anti-EMA the high sensitivity (98 and 95% respectively) and specificity (98 and 99%), moderate positive predicted values of (72 and 83%) and high negative predicted values (99 and 99%) make these autoantibodies useful markers for correctly detecting CD [25]. The inflammatory marker ferritin has also been used to tease out differences in patients with CD on a normal diet with those on a gluten-free diet. Patients with CD on normal diet have a significantly lower ferritin levels and higher soluble transferrin receptor [26, 27]. Children with CD can present with very subtle signs, therefore having a robust screening criteria is extremely important in aiding a difficult clinical diagnosis.

## Case Presentation

A six year and eleven month old girl of non-consanguineous Caucasian parents first presented with a two week history of fever, weight loss, arthralgia and maculopapular rash. She was born at term *via* Caesarean section with no neonatal concerns. Her medical history was fairly unremarkable, with mild eczema and croup and normal growth. There was no family history of autoimmune conditions. She was up-to-date with her immunizations. She was initially diagnosed with tonsillitis which was positive for Streptococcus on a throat swab. She remained unwell despite recurrent courses of antibiotics over a 6 week period. A comprehensive viral surveillance including Epstein Barr Virus (EBV), Cytomegalovirus (CMV), HIV and Hepatitis B using PCR on blood was negative on admission. She attended her local hospital due to persisting symptoms. She had no clinical features of arthritis or bowel disease. Serum inflammatory markers were prominent including C-Reactive Protein 135 mg/l (0-10 mg/l), thrombocytopenia with a platelet count of 64 × 10^9^/l (150–300 × 10^9^/l), raised alanine transaminase (ALT), 1038 IU/l (10–35 IU/l), raised lactate dehydrogenase (LDH), 8775 IU/L (420–750 IU/l), hyperferritinaemia, 71,378 μg/l (23–76 μg/l), hypofibrinogenaemia, 1 g/l (1.5–4.0 g/l). Her haemoglobin (Hb) and white cell count (WCC) were 80 g/L (115–155 g/l) and 5.1 × 10^9^/l (4.5–13.51 × 10^9^/l) respectively. She was referred for further investigation; her bone marrow biopsy showed occasional haemophagocytosis; flow cytometry perforin expression was present, there was normal granule release (CD107a) on activation of CD8 and NK cells and her CD56 + ve cells were normal (82.4%) compared to ﻿the control (71.5%). A basic auto-immune screen was negative (anti-neutrophil cytoplasmic antibodies (ANCA), rheumatoid factor, anti-nuclear antibodies (ANA), anti-double-stranded DNA (anti-dsDNA), and anti-cyclic citrullinated peptide (anti-CCP)). A presumed diagnosis of secondary HLH was made as she fulfilled HLH criteria without any genetic cause with normal granule release. She was treated with dexamethasone (8 weeks), etoposide (12 weeks), cyclosporine A (weaned from 7 months) as per HLH 2004 protocol. She made a good recovery, her biochemical markers normalised (ferritin 42 μg/l, CRP <5 mg/l, ALT 26 IU/ml, platelets 260 x 10^9^/l, Hb 120 g/l) and she remained well for 20 months.

She subsequently represented with a HLH relapse having been unwell for 2 weeks with headaches and lethargy and 5-days of fever. Due to her previous medical history she was screened for a relapse of secondary HLH. Serum ferritin level was 6702 μg/l and LDH 1002 u/l. Repeat bone marrow aspirate showed no evidence of haemophagocytosis. She continued to have quotidian fevers occurring in the evening and night for over a month with other similar symptoms to the initial presentation, arthralgia, a migratory erythematous rash, lethargy and significant weight loss of 2 kg. A skin biopsy showed neutrophilic leucocytoclastic vasculitis therefore she was further investigated by paediatric rheumatology for an underlying systemic condition with an angiogram which showed no specific evidence of vasculitis. Further auto-antibody screening detected a raised anti-tissue transglutaminase (anti-TTG) at 108U/ml (0–6.9U/ml) and positive anti-endomysial antibodies (anti-EMA) indicating a positive coeliac screen. Her HLA typing was positive for HLA-DQ2 but negative for HLA DQ8. She was too unwell to tolerate an endoscopy and jejunal biopsy. There had been no abdominal symptoms and there was no family history of Coeliac Disease. She commenced a gluten-free diet without any Disease Modifying Anti-Rheumatic Drugs (DMARDs) or biological agents, after which her symptoms dramatically improved with her energy levels rising to 90% of normal, however, the rash took longer to fade. Her biochemical markers also improved with her anti TTG level normalising to 4.3U/ml and her ESR reducing from 34 mm/h to 12 mm/h (<13 mm/h) within a month of a gluten free diet. She remained extremely sensitive to gluten, any exposure caused a reaction within a few hours with fevers lasting up to seven days. This suggested that the driving factor for her inflammatory process was due to Coeliac Disease. In light of her hypersensitivity and severe clinical condition, it was felt that a re-challenge with gluten was not appropriate.

Since establishing a gluten-free diet she remained well with no further episodes of MAS. However, after 3 years of commencing a gluten free diet she developed swelling of her joints and was diagnosed with arthritis, mainly affecting the left elbow, which required joint injections and treatment with methotrexate. The family have noted that her symptoms correlate with mistakenly eaten food containing gluten. At no point has she had any bowel symptoms. There was no active arthritis, morning stiffness or limping during either episode of MAS, making SoJIA unlikely. A jejunal biopsy has not been carried out as she has remained on a strict gluten-free diet. Care was provided by a multi-professional team of rheumatologists, gastroenterologists and haematologists.

## Discussion

Macrophage activation syndrome remains a serious condition which can be easily missed unless there is a high index of suspicion. The striking mortality associated with MAS makes it an important differential for patients with symptoms of systemic inflammation. Previous studies have indicated a relationship between MAS and auto-immune diseases in particular SoJIA, SLE, autoinflammatory (fever) syndromes and Kawasaki disease. There is evidence that Coeliac Disease is associated with other autoimmune conditions such as JIA, type I diabetes mellitus and thyroid disease [28–31]. This case report indicates that coeliac disease can trigger the pro-inflammatory process of macrophage activation.

The pathophysiology of both CD and MAS have not been fully established, therefore only a presumptive mechanism to connect the two conditions can be formed. The mucosal damage is not only caused by components of gluten but also directly by intraepithelial lymphocytes and other immune mediated cells including macrophages and natural killer cells [32]. Once these cells are activated the positive feedback to regulate them becomes disorganised, chaotic and invades the bone marrow leading to haemophagocytosis. The underlying question is what triggers the autoimmune process, and more importantly why does it become chaotic in some patients? The theoretical answer of a genetic predisposition, such as perforin or Munc-2 deficiency, followed by an environmental trigger needs more research and evaluation.

Coeliac Disease can present insidiously with symptoms differing between those under 3 years and those over 3 years old [33]. Symptoms of children under 3 years are predominantly gastrointestinal related (diarrhoea, constipation, abdominal pain and failure to thrive) whilst those over 3 years of age are generally extra-gastrointestinal, including screening of high risk children, abnormal blood results (iron deficiency anaemia, high transaminases) and other symptoms of short stature, alopecia and mood disorders [34]. This results in difficulty in recognising CD and potential delayed diagnosis [35]. Therefore, the challenge of diagnosing CD together with the strong association with other autoimmune diseases should prompt CD screening in rheumatological patients.

The recently updated 2016 classification criteria for MAS complicating SoJIA provides some assistance for the potential diagnosis of MAS complicating CD. The expert panel together with patient data found that 98.9% of patients with a confirmed diagnosis of MAS had fever at disease onset.[12] This has become essential in the diagnosis of MAS. In contrast, fever does not feature as a clinical symptom at diagnosis of CD [33, 36–38]. Patients with CD who present with prolonged fever of unknown origin should be evaluated for MAS by a thorough examination and measurement of biochemical markers as outlined by the HLH-2004 criteria [11]. Further validation of the 2016 classification criteria for MAS complicating SoJIA may provide a better platform for diagnosis of MAS in autoimmune disease in the future.

## Conclusion

Macrophage activation syndrome is a severe autoimmune disease of chaotic immune dysregulation that requires early aggressive treatment. We present the first case report that links macrophage activation syndrome to Coeliac Disease and highlights the challenge of diagnosing Coeliac Disease with unusual features such as associated prolonged fever. In managing patients with macrophage activation syndrome, clinicians should have a low threshold for screening children for other autoimmune diseases including Coeliac Disease.

## Abbreviations

ALT: Alanine transaminase
ANA: Anti-nuclear antibodies
ANCA: Anti-neutrophil cytoplasmic antibodies
anti-CCP: Anti-cyclic citrullinated peptide
anti-dsDNA: Anti-double-stranded DNA
anti-tTG: Anti-tissue transglutaminase
CD: Coeliac Disease
DMARDs: Disease Modifying Anti-Rheumatic Drugs
EMA: Anti-endomysium antibodies
fHLH: Familial Haemophagocytic lymphohistiocytosis
HLH: Haemophagocytic lymphohistiocytosis
JIA: Juvenile Idiopathic Arthritis
MAS: Macrophage activation syndrome
SLE: Systemic Lupus Erythematosus
SoJIA: Systemic-Onset Juvenile Idiopathic Arthritis
